# Advances in understanding the renin-angiotensin-aldosterone system (RAAS) in blood pressure control and recent pivotal trials of RAAS blockade in heart failure and diabetic nephropathy

**DOI:** 10.12688/f1000research.9692.1

**Published:** 2017-03-21

**Authors:** Lama Ghazi, Paul Drawz

**Affiliations:** 1 Division of Renal Disease and Hypertension, Department of Medicine, University of Minnesota, Minnesota, MN, USA

**Keywords:** renin-angiotensin-aldosterone system, hypertension, blood pressure control, anti-hypertensive drugs, blocking agents

## Abstract

The renin-angiotensin-aldosterone system (RAAS) plays a fundamental role in the physiology of blood pressure control and the pathophysiology of hypertension (HTN) with effects on vascular tone, sodium retention, oxidative stress, fibrosis, sympathetic tone, and inflammation. Fortunately, RAAS blocking agents have been available to treat HTN since the 1970s and newer medications are being developed. In this review, we will (1) examine new anti-hypertensive medications affecting the RAAS, (2) evaluate recent studies that help provide a better understanding of which patients may be more likely to benefit from RAAS blockade, and (3) review three recent pivotal randomized trials that involve newer RAAS blocking agents and inform clinical practice.

## New and existing anti-hypertensive drugs affecting the renin-angiotensin-aldosterone system

**Figure 1.**
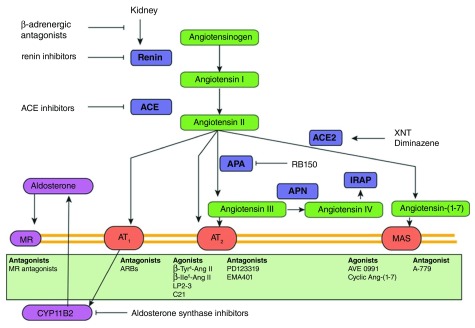
New and existing drugs interfering with the renin-angiotensin (Ang) system cascade. Classically, interference occurs at the level of renin, angiotensin-converting enzyme (ACE), the Ang II type 1 (AT _1_) receptor (R), or the mineralocorticoid receptor (MR), with renin inhibitors, ACE inhibitors, AT _1_ receptor blockers (ARBs), or MR antagonists. Novel enzyme inhibitors now target aminopeptidase A (APA), which generates Ang III (=Ang-[1–7]) from Ang II (=Ang-[1–8]), or aldosterone synthase (CYP11B2). Activators of ACE2 (XNT and diminazene), which generates Ang-(1–7) from Ang II, were recently found to act equally well in ACE2 knockout animals, thus questioning their mechanism of action. Numerous agonists for both the AT _2_ receptor and Mas receptor are being developed. Aminopeptidase N (APN) degrades Ang III to Ang IV (=Ang-[3–8]), which may act on the AT _4_ receptor, also known as insulin-regulated aminopeptidase (IRAP). Figure reprinted with permission of the American Heart Association ^57^.

There are well-established drugs that interfere with the renin-angiotensin-aldosterone system (RAAS) at several sites, including (1) angiotensin-converting enzyme inhibitors (ACEIs), (2) angiotensin II type I (AT _1_) receptor blockers (ARBs), (3) direct renin inhibitors (DRIs), (4) mineralocorticoid receptor antagonists (MRAs), and even (5) beta blockers, the last of which may be considered partial inhibitors ( Figure 1) ^1– 4^. In this section, we briefly review trials demonstrating benefits of ACEIs/ARBs and then discuss recent advances and newer agents that block the RAAS at different sites.

### Angiotensin-converting enzyme inhibitors and angiotensin II type I receptor blockers

ACEIs and ARBs have been the cornerstone of RAAS inhibition for years and are key therapeutic options in patients with hypertension (HTN), reducing cardiovascular morbidity and mortality and improving renal outcomes. In the HOPE (Heart Outcomes Prevention Evaluation) ^5^, MICRO-HOPE (The Microalbuminuria, Cardiovascular, and Renal Outcomes in HOPE) ^6^, EUROPA (European Trial on Reduction of Cardiac Events with Perindopril in Stable Coronary Artery Disease) ^7^, SOLVD (Studies of Left Ventricular Dysfunction) ^8^, and Captopril Prevention Project ^9^ studies, ACEIs were beneficial in reducing rates of death, myocardial infarction, stroke, coronary revascularization, cardiac arrest, and heart failure, complications related to diabetes and heart failure. Both the RENAAL ^10^ and IDNT ^11^ trials demonstrated a renoprotective effect of RAAS inhibition in nephropathy due to diabetes. ACEIs and ARBs are considered to be equally beneficial on the basis of studies such as ONTARGET ^12^, which compared telmisartan and ramipril, and DETAIL ^13^, which compared telmisartan with enalapril and found no difference in progression of diabetic nephropathy.

Azilsartan, a novel ARB, has been shown to have superior efficacy in reducing blood pressure (BP) when compared with olmesartan or valsartan ^14– 16^. Azilsartan 80 mg reduces BP more efficaciously than the optimal and highest tolerated dosage of both olmesartan (40 mg) and valsartan (320 mg) using 24-hour ambulatory BP monitoring and without increase in adverse effects ^14^. A recent analysis of a German registry confirmed these results; among 3,849 patients with essential HTN, 61% of patients who initiated therapy with azilsartan achieved a BP target of less than 140/90 mmHg compared with 56% of those who were started on an ACEI ^17^. Azilsartan may be more effective at lowering BP because of its more potent ability to block AT _1_ receptors ^18^. However, only a prospective, randomized, dose-escalation study can truly test whether azilsartan is superior to other ARBs at lowering BP.

### Direct renin inhibitors

Renin secretion, a rate-limiting step, is the first step of the RAAS cascade. Renin has a unique specificity for its substrate angiotensin. Inhibition of renin provides an attractive option to inhibit the RAAS from its origin. The development of DRI started more than 30 years ago ^19^, but there were issues with potency, bioavailability, and cost. Currently, aliskiren is the only approved DRI for use in HTN ^20^, and a significant BP reduction has been demonstrated in patients with essential HTN ^21, 22^. Aliskiren is well tolerated and has a similar dose-dependent BP reduction in hypertensive patients as ARBs and a safety profile similar to that of placebo ^23, 24^.

However, several recent studies have shown either no benefit or even harmful effects of aliskiren in certain populations. The ALTITUDE (Aliskiren Trial in Type 2 Diabetes Using Cardio-Renal Endpoints) ^25^ trial randomly assigned patients with type 2 diabetes and chronic kidney disease (CKD) or with cardiovascular disease (or with all three) already receiving an ACEI or ARB to aliskiren or placebo. Although there was a lower BP in the aliskiren arm, there was no reduction in the primary composite outcome, which included cardiovascular and renal events and mortality (hazard ratio [HR] 1.08, 95% confidence interval [CI] 0.98–1.20). In the ATMOSPHERE ^26^ trial (Aliskiren Trial to Minimize Outcomes in Patients with Heart failure), the addition of aliskiren to enalapril in patients with chronic heart failure was not associated with reduction in adverse outcomes (see “Recent clinical trials” section for details). Similarly, no improvement in coronary atherosclerosis in pre-hypertensive patients (AQUARIUS) ^27^ or improvement in cardiovascular outcomes in patients hospitalized with heart failure (ASTRONAUT) ^28^ was seen with aliskiren compared with placebo. Given the lack of demonstrated benefit and increased rates of adverse events such as hyperkalemia, hypotension, and renal impairment as seen in ASTRONAUT when combined with ACEIs or ARBs, the current use of aliskiren is limited ^28^.

### Mineralocorticoid receptor antagonists

MRAs competitively inhibit mineralocorticoid receptors and decrease the number of epithelial sodium channels in the distal renal tubule ^29^. Spironolactone, an MRA, has long been used for the treatment of HTN; however, its non-specificity for mineralocorticoid receptors manifests as anti-androgenic and progestational effects ^30, 31^. Spironolactone was found to be the most effective add-on anti-hypertensive drug when compared with doxazosin and bisoprolol in treating resistant HTN in the PATHWAY-2 trial ^32^. This trial supports the important role of sodium retention in resistant HTN. Eplerenone, an MRA with lower affinity to progesterone and androgen receptors than spironolactone ^29^, has been shown to be efficacious even using ambulatory monitoring studies ^33^ and safe in the management of HTN. When compared with enalapril ^34^, losartan ^35^, and amlodipine ^36^, eplerenone monotherapy was as efficacious in treating HTN.

Currently, third- and fourth-generation MRAs are being developed to have the potency of spironolactone and the selectivity of eplerenone ^37^. Finerenone, a novel non-steroidal MRA, has a greater affinity to the mineralocorticoid receptor than does eplerenone ^38^ and greater selectivity than does spironolactone ^39^. The Mineralocorticoid Receptor Antagonist Tolerability Study (ARTS) showed that finerenone (2.5–10 mg per day), in patients with CKD and albuminuria, decreased albuminuria with lower rates of hyperkalemia compared with spironolactone ^40^. With the recent development of patiromer, a non-absorbed potassium binder, a safe option for the treatment of chronic hyperkalemia is available, maintaining and using RAAS inhibitors including spironolactone, in CKD and heart failure ^41^. Patiromer has also been shown to decrease potassium and aldosterone levels in patients with CKD and hyperkalemia on RAAS inhibitors independent of renin activity ^42^. The recent ARTS-DN (Mineralocorticoid Receptor Antagonist Tolerability Study–Diabetic Nephropathy) study demonstrated greater reduction in albuminuria with the addition of finerenone to ACEI or ARB in patients with diabetic nephropathy compared with placebo (see “Recent clinical trials” section for details) ^43, 44^.

### Aldosterone synthase inhibitors

Another way of blocking the effects of mineralocorticoid receptor activation is to inhibit aldosterone formation. LCI699 is a potent first-in-class aldosterone synthase inhibitor. In patients with primary aldosteronism, LCI699 (up to 1.0 mg twice a day) caused modest reduction in 24-hour systolic BP (SBP) and office SBP compared with placebo ^45^. One mg of LCI699 was not superior to eplerenone 50 mg in reducing ambulatory SBP in patients with stage 1 or 2 HTN ^46^. LCI699 significantly lowered office and ambulatory BP in patients with primary HTN, but 20% of the patients on LCI699 developed blunted cortisol release ^46^. Because of this non-specificity, the development of LCI699 has been stopped in favor of developing more specific inhibitors.

### Aminopeptidase A inhibitor

Functional RAAS may be present in the brain ^47^, and several animal models have demonstrated that hyperactivity of brain RAAS may contribute to HTN ^48, 49^. Brain aminopeptidase A (APA) converts angiotensin II to angiotensin III. APA inhibition by EC33 has been shown to be a novel anti-hypertensive agent in animals ^50– 52^. QGC001, an orally active prodrug of EC33, was tolerated in healthy normotensive human males but with no changes in renin, aldosterone, BP, or heart rate ^53– 56^. This study demonstrates that QGC001, the first drug in its class, can be safely administered to humans, but further studies are required to assess the safety and efficacy of QGC001 in patients with HTN. Additional research on novel ways to block the RAAS, including development of prorenin blockade, gene- and vaccine-based strategies, and targeting ACE2, is being developed but has not yet translated into the clinic ^57, 58^.

## Predictors of renin-angiotensin-aldosterone system blockade response in patients with hypertension

In this section, we will review recent advances in understanding characteristics that may predispose patients to a greater response to RAAS blockade.

### Plasma renin activity

Patients with high plasma renin activity (PRA) respond well to RAAS blockers, some of which suppress renin release ^59– 62^. For example, patients with elevated renin had a greater BP-lowering response to atenolol, an agent that suppresses renin release, than a diuretic ^60, 61^. In contrast, in patients with low renin, anti-renin drugs (including aliskiren) may exert a pressor effect ^61, 63^.

Using PRA levels as a treatment strategy is currently being tested as a way to improve BP control ^64, 65^. Egan *et al*. showed that, in patients with uncontrolled HTN, ambulatory PRA provides information on the extracellular fluid and plasma volume ^64^. In their trial, a “renin test-guided therapeutic (RTGT) algorithm” targeted RAAS blockers to patients with high renin. The RTGT resulted in greater reduction in SBP compared with clinical HTN specialist care (–29.1 ± 3.2 mmHg versus –19.2 ± 3.2 mmHg; *P* = 0.003). Therefore, it would seem logical to treat hypertensive patients with high PRA with anti-renin-angiotensin drugs as a first choice ^64^. Although targeting RAAS blockade to patients with high PRA may be an effective BP-lowering strategy, further studies on the effect of RTGT treatment on risk for clinically important end-points are needed before this strategy can be recommended.

### Salt

Salt plays an important role in BP response to RAAS blockers, although the exact mechanism remains to be elucidated. High salt intake causes volume expansion and BP elevation, which leads to pressure-dependent tissue injury, including renal injury ^66– 70^. RAAS inhibitors reverse the salt-induced renal injury in spontaneous hypertensive rats ^71– 73^.

Kobori *et al*. showed that a high-salt diet decreased PRA and enhanced kidney angiotensinogen levels in Dahl-sensitive rats, contributing to HTN ^74^. An increase in angiotensin II contributes to activation of the mineralocorticoid receptor-dependent intracellular signaling pathway, perhaps via induction of reactive oxygen species, and it has been proposed that mineralocorticoid receptor activation can occur independently of aldosterone in salt-sensitive HTN ^75– 77^. This has recently been demonstrated in a study of patients with resistant HTN. Ghazi *et al*. examined predictors of BP response to spironolactone in a retrospective analysis of 79 patients with resistant HTN ^78^. Patients with high urinary sodium excretion (200 mEq/24 hours) had a significantly greater BP reduction with spironolactone compared with patients with a lower excretion (<200 mEq/24 hours) ( *P* = 0.008). After potential confounders, primary aldosteronism, serum aldosterone concentration, and serum potassium were controlled for, 24-hour urinary sodium excretion remained a significant, independent predictor ( *P* = 0.02) of a favorable BP response, defined as an at least 10 mmHg reduction in office SBP. Despite its limitations, including its retrospective design, not using 24-hour ambulatory BP monitoring, and non-generalizability to the general HTN population with controlled BP, it is the first study to suggest the benefit of MRA in counteracting the effects of high-sodium diet in patients with HTN, irrespectively of their aldosterone status ^78^. Given the difficulty in modifying patients’ dietary habits, elevated 24-hour urine sodium excretion may be used to identify patients who are more likely to be responsive to spironolactone.

### Ethnicity

It is well known that African-Americans (AAs) have a different response to RAAS blockers when compared with whites. This might be due to several mechanisms, including salt sensitivity, low renin, and high aldosterone levels, which may be interrelated ^79– 83^. In ALLHAT (Antihypertensive and Lipid-Lowering Treatment to Prevent Heart Attack Trial) and the blood pressure-lowering arm of the Anglo-Scandinavian Cardiac Outcomes Trial (ASCOT-BPLA), ACEIs were less effective in reducing BP in AAs compared with whites; however, no racial difference was observed in those randomly assigned to a diuretic ^84– 86^. This effect is not limited to ACEIs. In a randomized placebo-controlled study in AAs with low renin and poorly controlled HTN, both spironolactone and amiloride lowered BP similarly in AAs and whites ^87^. However, AAs had a significantly better response to eplerenone than to losartan (ARB), whereas no difference in response between these two agents was found in whites ^88^. In patients with heart failure treated with spironolactone, AAs exhibit less hyperkalemia compared with whites when treated with MRAs ^89^.

A recent study on patients from New York City’s Health and Hospitals Corporation compared ACEI efficacy to calcium channel blocker (CCB), thiazide diuretics, and beta blockers in AAs. It included a cohort of 25,564 propensity score-matched hypertensive AA patients. ACEIs were associated with a higher risk of primary outcome (myocardial infarction, stroke, and heart failure) compared with CCB (4,506 matched pairs; HR 1.45, 95% CI 1.19–1.77; *P* = 0.0003), and a higher risk for primary outcome was observed when ACEIs were compared with thiazide diuretics in AAs (5,337 matched pairs; HR 1.65, 95% CI 1.33–2.05; *P* < 0.0001) ^90^. A meta-analysis of 13 different trials in the USA and Europe showed that SBP and diastolic BP reduction with ACEI monotherapy was consistently lower among AAs than among whites ^91^. Therefore, as recommended by the members appointed to the Eighth Joint National Committee (JNC 8), initial anti-hypertensive therapy for the AA population should include a thiazide or CCB.

### Sex

The RAAS is affected by sex hormones and plays a role in sex-related differences in BP; however, there are no specific guidelines that suggest sex-specific treatment ^57, 92– 94^. Estrogen attenuates the vasoconstrictor effect of the RAAS by decreasing renin, angiotensin, and nitric oxide synthase, but this attenuation will decrease once women reach menopause ^57, 95, 96^. Irbesartan, for example, has a greater BP-lowering effect in pre-menopausal women than in men ^97^. In a retrospective observational study of congestive heart failure (CHF), women had a better survival with ARBs than with ACEIs whether they were hypertensive or not ^98^. In hypertensive men, their survival was comparable using an ARB or an ACEI. In non-hypertensive men, ACEIs were associated with better survival.

More recently, the Identification of the Determinants of the Efficacy of Arterial blood pressure Lowering drugs (IDEAL) trial set out to identify office SBP response to both an ACEI (perindopril) and a diuretic (indapamide) in untreated hypertensive patients (SBP ≥140 and <180 mmHg) between 25 and 70 years of age ^99^. For the 112 patients included in this cross-over study, the average BP responses were 7.2/4.0 mmHg for indapamide and 6.6/4.2 mmHg for perindopril. The response among women, without distinction among pre- or post-menopausal, was almost two times greater than in men for indapamide (mean effect of –11.5 mmHg for women versus –4.8 mmHg for men; *P* <0.001) and for perindopril (mean effect of –8.3 mmHg for women versus –4.3 mmHg for men; *P* = 0.015). Subgroup analysis of the response to indapamide by age revealed that an association was present in women and that there was an increase of the response by 3 mmHg for every 10 years of age ( *P* = 0.024). Therefore, both age and sex were important determinants of BP response in the IDEAL population.

BP response to various RAAS blockades is affected by renin, salt, gender, ethnicity, and even age. However, all of those factors are likely interrelated. For instance, AAs have lower renin activity than do whites and have been shown to respond better to atenolol ^62, 86^. Patients on a low-salt diet have higher renin activity ^57^.

## Recent clinical trials

In this final section, we review a few recent pivotal trials of RAAS blockade.

### ATMOSPHERE trial

RAAS blockade reduces the risk of death among patients with heart failure with reduced ejection fraction ^8^. Dual blockade with an ACEI and aliskiren reduced N-terminal prohormone of brain natriuretic peptide (NT-proBNP). Therefore, the ATMOSPHERE trial ^26^ randomly assigned patients in a 1:1:1 fashion to aliskiren 300 mg once daily (n = 2,340), enalapril 5 or 10 mg twice daily (n = 2,336), or enalapril 5 or 10 mg twice daily + aliskiren 300 mg once daily (n = 2,340), and patients were followed-up for 36.6 months (interquartile range of 22.4 to 52.2). ATMOSPHERE included patients at least 18 years of age with chronic symptomatic heart failure (New York Heart Association functional class II–IV), left ventricular ejection fraction of not more than 35%, and an estimated glomerular filtration rate (eGFR) of at least 40 mL/minute per 1.73 m ^2^.

The Clinical Trials Facilitation Group of the Heads of Medicines Agencies in Europe requested discontinuation of ATMOSPHERE study drugs in diabetic patients following the results of the ALTITUDE and ASTRONAUT trials. Overall, there was no difference in the primary outcome, cardiovascular death, or CHF hospitalization for aliskiren + enalapril versus enalapril (32.9% versus 34.6%; HR 0.93, 95% CI 0.85–1.03; *P* = 0.17 for superiority) or for aliskiren versus enalapril (33.8% versus 34.6%; HR 0.99, 95% CI 0.90–1.10; *P* = 0.91 for superiority) ( *P* = 0.018 for non-inferiority; not significant per pre-specified threshold). There were no differences in most of the secondary outcomes, but combination therapy was associated with increased risk for the composite renal outcome (HR 2.2, 95% CI 1.2–3.8). Combination therapy was also associated with increased risk for hypotension and hyperkalemia. In all, ATMOSPHERE indicates that aliskiren is not superior to enalapril either as monotherapy or as combination therapy in patients with heart failure with reduced ejection fraction.

### ARTS-DN trial

Finerenone is a non-steroidal MRA that is equally efficacious as spironolactone at reducing BNP and albuminuria in patients with CKD and heart failure with reduced ejection fraction but has the added benefit of lower incidence of hyperkalemia ^40^. ARTS-DN was a multicenter, randomized trial designed to compare the effects of finerenone 1.25 to 20 mg once daily with placebo when added to the standard of care with an RAAS blocker. Patients with type 2 diabetes mellitus, persistent albuminuria (urine albumin-to-creatinine ratio [UACR] of at least 30 mg/g), and eGFR of more than 30 mL/minute per 1.73 m ^2^ receiving treatment with the minimum recommended dosage of an RAAS blocker prior to the screening visit were included.

The primary outcome was the UACR at day 90 compared with baseline. Finerenone reduced UACR in a dose-dependent manner. The placebo-corrected geometric mean UACRs at day 90 to baseline in finerenone 7.5, 10, 15, and 20 mg groups were 0.79 (90% CI 0.68–0.91; *P* = 0.004), 0.76 (90% CI 0.65–0.88; *P* = 0.001), 0.67 (90% CI 0.58–0.77; *P* <0.001), and 0.62 (90% CI 0.54–0.72; *P* <0.001), respectively. The incidences of an eGFR decrease of at least 40% at any time after baseline were similar in the placebo and finerenone groups, and decreases in eGFR noted in the finerenone groups were reversible 30 days after completion of treatment. There was no reported difference in serious adverse events. This trial demonstrated that, in patients with diabetic nephropathy receiving an ACEI or ARB, adding finerenone compared with placebo improved UACR without an increased risk of hyperkalemia. Longer-term studies are needed to determine whether the addition of finerenone is renoprotective.

### VA NEPHRON-D trial

The goal of the VA NEPHRON-D (Veterans Affairs Nephropathy in Diabetes) trial ^100^ was to evaluate whether combination treatment with ACEI (lisinopril) and ARB (losartan) compared with ARB alone in patients with diabetic nephropathy slows the progression of CKD. Patients with diabetes, eGFR of 30.0–89.9 mL/minute per 1.73 m ^2^, and a UACR of more than 300 mg/g were included. After 2.2 years, the primary outcome of decrease in eGFR, end-stage renal disease, or death occurred in 18.2% of participants in the combination ACEI/ARB group versus 21.0% of the ARB group (HR in combination group of 0.88, 95% CI 0.7–1.12; *P* = 0.30). There was increased risk for adverse events in the combination group versus ARB alone, including acute kidney injury (18.0% versus 11.0%; *P* <0.001) and hyperkalemia (9.9% versus 4.4%; *P* <0.001). The increased risk for adverse events led to early termination of the trial. Combined with the results of ONTARGET, VA NEPHRON-D confirms that there is no benefit but an increased risk of adverse events from dual inhibition from RAAS in high-risk patients.

## Conclusions

Despite the large number and varying mechanisms of action of existing RAAS-blocking agents, complications such as hyperkalemia and acute kidney injury limit their use. Newer agents that are more specific, such as finerenone, are being developed and may provide the benefits of older RAAS-blocking agents with fewer complications and adverse effects. However, lessons learned from newer agents such as aliskiren indicate that long-term studies with hard end-points are required because outcomes from short-term trials with surrogate outcomes (for example, albuminuria and BNP) may not be consistent with long-term trials with hard clinical end-points. Insight from mechanistic studies and retrospective analyses will inform the design of these trials to target patients most likely to benefit from RAAS blockade, further refining our approach of the use of RAAS blockers in the treatment of HTN.
